# *N*-Butanol Subfraction of *Brassica Rapa* L. Promotes Reactive Oxygen Species Production and Induces Apoptosis of A549 Lung Adenocarcinoma Cells via Mitochondria-Dependent Pathway

**DOI:** 10.3390/molecules23071687

**Published:** 2018-07-11

**Authors:** Adila Aipire, Qiuyan Chen, Shanshan Cai, Jinyu Li, Changshuang Fu, Tianlei Ying, Jun Lu, Jinyao Li

**Affiliations:** 1 Xinjiang Key Laboratory of Biological Resources and Genetic Engineering, College of Life Science and Technology, Xinjiang University, Urumqi 830046, Xinjiang, China; kaskas999@163.com (A.A.); m15276567620_1@163.com (Q.C.); 18699927521@163.com (S.C.); fu_ss1220@163.com (C.F.); 2 College of Life Science, Xinjiang Normal University, Urumqi 830054, Xinjiang, China; 3 Key Laboratory of Medical Molecular Virology of MOE/MOH, Shanghai Medical College, Fudan University, 130 Dong An Road, Shanghai 200032, China; tlying@fudan.edu.cn; 4 School of Science, and School of Interprofessional Health Studies, Faculty of Health & Environmental Sciences, Auckland University of Technology, Private Bag 92006, Auckland 1142, New Zealand; jun.lu@aut.ac.nz

**Keywords:** *Brassica rapa* L., antitumor, reactive oxygen species, apoptosis, cell cycle arrest, migration

## Abstract

*Brassica rapa* L., an edible and medical vegetable, has been traditionally used in Uyghur folk medicine to treat coughs and asthma in the Xinjiang Uygur Autonomous Region, China. In this study, we prepared an *n*-butanol subfraction of *B*. *rapa* L. (BRBS) and investigated the anti-tumor effect on A549 lung adenocarcinoma cells. The proliferation of A549 cells was significantly inhibited by BRBS treatment in a dose- and time-dependent manner. BRBS significantly induced cell cycle arrest and apoptosis in A549 cells through increased reactive oxygen species (ROS) production and mitochondrial dysfunction characterized by a reduction in mitochondrial membrane potential and the release of cytochrome c, which promoted caspase-3 and poly(ADP-ribose) polymerase processing. Moreover, BRBS significantly suppressed the migration of A549 cells in vitro. These results suggest that BRBS inhibited A549 cell proliferation through increased ROS production and the mitochondria-dependent apoptosis pathway. Consequently, BRBS might be a potential candidate for the treatment of lung cancer.

## 1. Introduction

Lung cancer is one of the most common cancers and the leading cause of cancer-related death worldwide [1]. In China, lung cancer is the most common cancer with around 733,300 new cases and 610,200 deaths in 2015 [2]. Although different strategies including chemotherapy, radiotherapy, targeted therapy and immunotherapy have been developed to treat lung cancer [3,4,5], the overall five-year survival rate is still lower than 18% [6]. Therefore, it is necessary to explore new drugs for the treatment of lung cancer.

Recently, a number of studies have been focused on the anti-tumor effects of natural products, especially traditional Chinese medicine [7,8,9,10]. *Brassica* vegetables belong to the Brassicaceae family and are cultivated in many countries. They contain various bioactive components including glucosinolates, carotenoids, tocopherols, ascorbic acid and phenolic compounds, and show both antioxidant and antitumor activities [11,12,13,14]. *Brassica rapa* L. has been used in Uyghur folk medicine to treat coughs and asthma for a long time in the Xinjang Uygur Autonomous Region, China [15]. More and more bioactive components including polysaccharides, phenolics, flavonoids and ascorbic acid have been isolated and identified from *B. rapa* L., which show different biological functions such as immunostimulation, anti-inflammation, anti-allergy and antioxidant [15,16,17,18,19,20]. However, the anti-tumor effects of *B. rapa* L. are yet to be investigated. In this study, we prepared the *n*-butanol subfraction of *B. rapa* L. (BRBS) and investigated the anti-tumor effect on A549 lung cancer cells. Our results showed that BRBS could induce apoptosis and cell cycle arrest in A549 cells through ROS generation and the mitochondria-dependent pathway.

## 2. Results

### 2.1. BRBS Inhibits the Proliferation of A549 Cells

**Figure 1:**
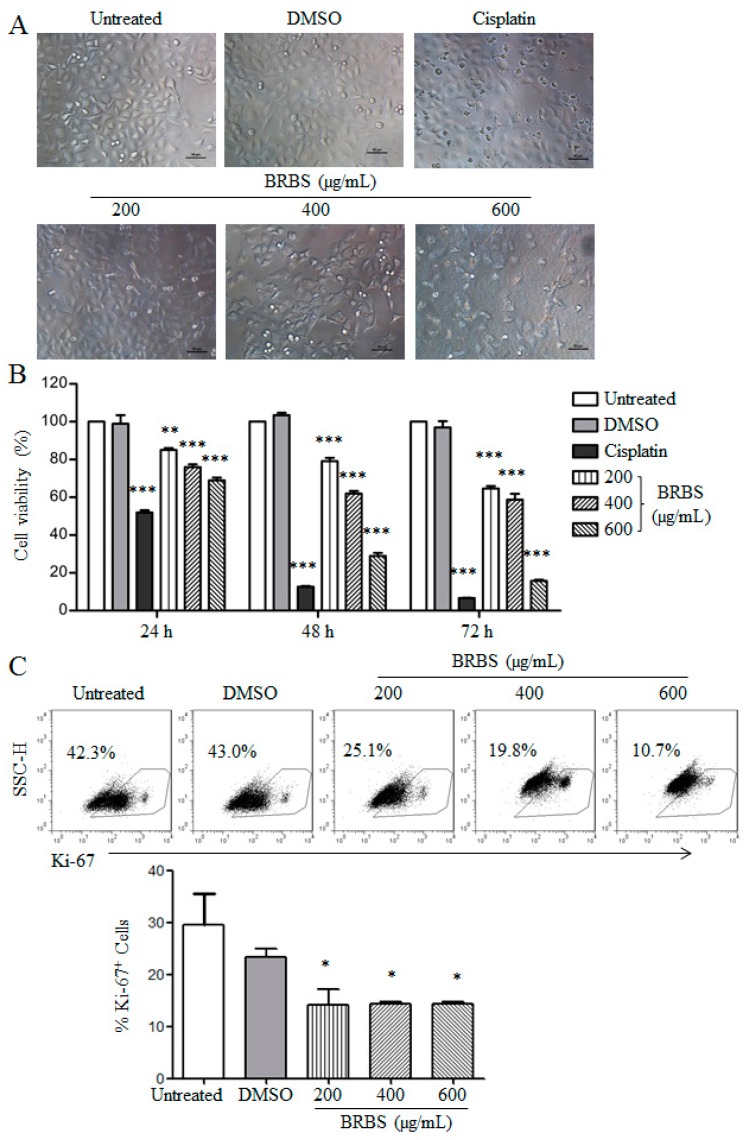
Effect of the *n*-butanol subfraction of *B. rapa* L. (BRBS) on morphology and proliferation of A549 cells (**A**) A549 cells were treated with BRBS for 24 h and observed under an inverted microscope. Scale bar = 50 µm; (**B**) Cells were treated with BRBS for 24, 48 and 72 h and cell viability was measured using an MTT assay. Data are expressed as Mean ± SEM (n = 6); (**C**) The expression of Ki-67 in A549 cells treated with BRBS for 24 h measured by flow cytometry. * *p* < 0.05; ** *p* < 0.01; *** *p* < 0.001 compared to Untreated.

To detect the anti-tumor activity of BRBS, we first examined the morphological changes of A549 cells upon treatment with various doses (200, 400 and 600 µg/mL) of BRBS for 24 h. After BRBS treatment, cells became round and shrunken and cell number also decreased compared with untreated cells, as shown in Figure 1A. Next, the viability of A549 cells was determined using an MTT assay upon BRBS treatment for 24, 48 and 72 h. The results showed that BRBS significantly reduced the viability of A549 cells in a dose- and time-dependent manner, as shown in Figure 1B. The IC_50_ values of BRBS at 24, 48 and 72 h were 1519, 483.2 and 429.4 µg/mL respectively. Finally, the proliferation of A549 cells was analyzed by Ki-67 staining, which is correlated with disease recurrence and progression in malignancies [21]. Consistently, BRBS significantly decreased the frequencies of Ki-67^+^ cells (Figure 1C), indicating that the proliferation of A549 cells was suppressed. These data show that BRBS inhibits the growth of A549 cells.

### 2.2. BRBS Induces Apoptosis and Cell Cycle Arrest in A549 Cells

**Figure 2:**
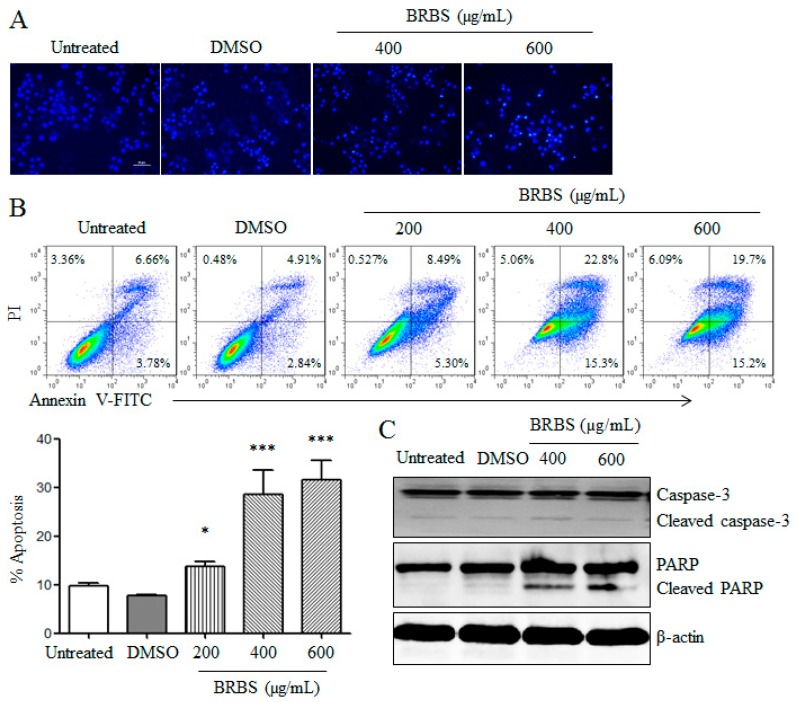
BRBS induces apoptosis of A549 cells. Cells were treated with BRBS for 24 h (**A**) Cells were stained with Hoechst 33258 and observed under an inverted fluorescence microscope. Scale bar = 50 µm; (**B**) Cells were stained with Annexin V-FITC/PI and samples were analyzed by flow cytometry. * *p* < 0.05; *** *p* < 0.001 compared to Untreated; (**C**) Protein samples of A549 cells were prepared and the levels of caspase-3 and poly(ADP-ribose) polymerase (PARP) were detected by Western blot.

We investigated whether BRBS inhibited the growth of A549 cells through induction of apoptosis. After BRBS treatment for 24 h, A549 cells were stained with Hoechst 33258 and the nuclear morphology was observed by inverted fluorescence microscope which displayed apoptotic characteristics such as chromatin condensation and fragmentation, as shown in Figure 2A. Subsequently, apoptosis of A549 cells was detected by flow cytometry. Compared with the control groups, BRBS treatment significantly increased the frequencies of apoptotic A549 cells (Annexin V^+^PI^−^ and Annexin V^+^PI^+^) in a dose-dependent manner, as shown as Figure 2B.

Activation of the caspase cascade and cleavage of its substrates such as poly(ADP-ribose) polymerase (PARP) have been used as a hallmark of apoptosis [22,23]. The protein levels were detected by Western blot after BRBS treatment for 24 h. As shown in Figure 2C, BRBS treatment promoted the cleavage of caspase-3 and PARP, indicating that BRBS induced cell apoptosis.

**Figure 3:**
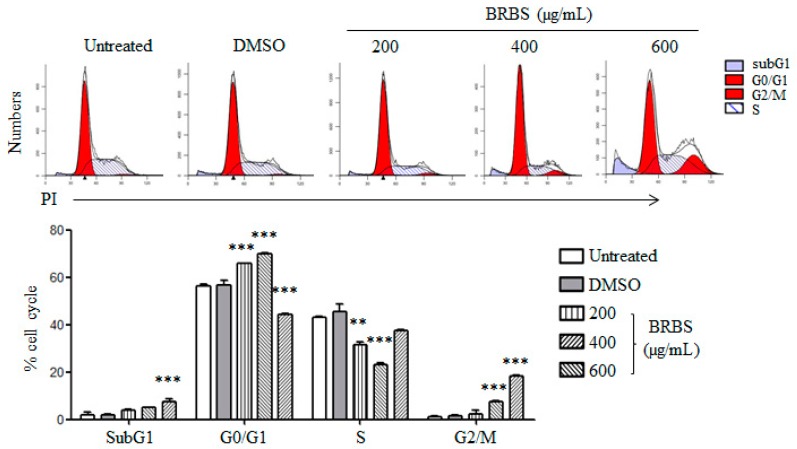
BRBS induces cell cycle arrest in A549 cells. Cells were treated with BRBS for 24 h and stained with propidium iodide (PI). Samples were analyzed by flow cytometry. ** *p* < 0.01; *** *p* < 0.001 compared to Untreated.

Following this, we further investigated the effect of BRBS on cell cycle distribution of A549 cells. After treatment with BRBS for 24 h, A549 cells were stained with propidium iodide (PI) and cell cycle distribution was analyzed by flow cytometry. As shown in Figure 3, the proportion of A549 cells at G0/G1 phase increased from 56.12% in untreated cells to 70.32% after treatment with 400 µg/mL BRBS. Furthermore, the proportion of A549 cells at G2/M phase increased from 0.91% in untreated cells to 18.88% after treatment with 600 µg/mL BRBS, suggesting that BRBS induced cell cycle arrest. This is consistent with the results of the MTT assay and Ki-67 staining. We also observed an accumulation of cells at subG1 phase that was directly related to the apoptosis induced by BRBS. These results suggest that BRBS inhibited the growth of A549 cells through induction of apoptosis and cell cycle arrest.

### 2.3. BRBS Reduces Mitochondrial Membrane Potential (MMP) of A549 Cells

**Figure 4:**
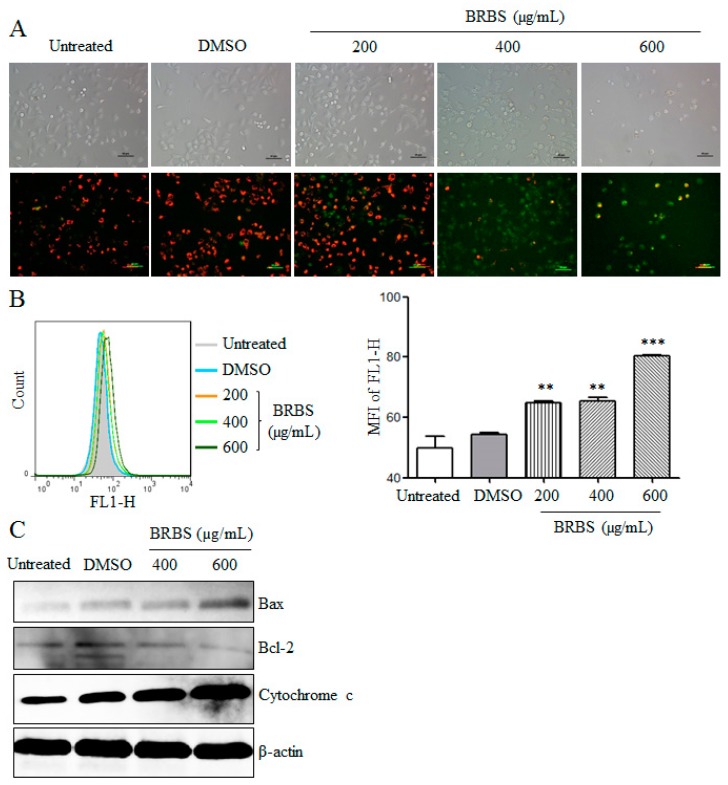
BRBS reduces the mitochondrial membrane potential (MMP) of A549 cells. Cells were treated with BRBS for 24 h and stained with JC-1. Samples were analyzed by inverted fluorescence microscope (Scale bar = 50 µm) (**A**) and flow cytometry (**B**). ** *p* < 0.01; *** *p* < 0.001 compared to Untreated. (**C**) Protein samples of A549 cells were prepared and the levels of Bax, Bcl-2 and cytochrome c were measured by Western blot.

Traditional Chinese medicine can induce tumor cell apoptosis through the mitochondria-dependent pathway [24,25]. We measured the MMP of A549 cells after BRBS treatment for 24 h. As shown in Figure 4A, A549 cells were observed by inverted fluorescence microscope after staining with JC-1 dye. The untreated cells showed red fluorescence but the BRBS treated cells showed increased green fluorescence in a dose-dependent manner. The fluorescence intensity of JC-1 dye was also analyzed by flow cytometry. The green fluorescence intensity was significantly increased upon BRBS treatment in a dose-dependent manner, as shown in Figure 4B, suggesting that MMP was reduced. Consistently, the level of cytochrome c was significantly increased in the cytoplasm of cells upon BRBS treatment, as shown in Figure 4C. We also observed that the expressions of Bax and Bcl-2 were upregulated and downregulated respectively, by BRBS treatment, also shown in Figure 4C, which may have caused the decrease of MMP. These results indicate that BRBS induced apoptosis of A549 cells through the mitochondria-dependent pathway.

### 2.4. BRBS Increases ROS Production in A549 Cells

**Figure 5:**
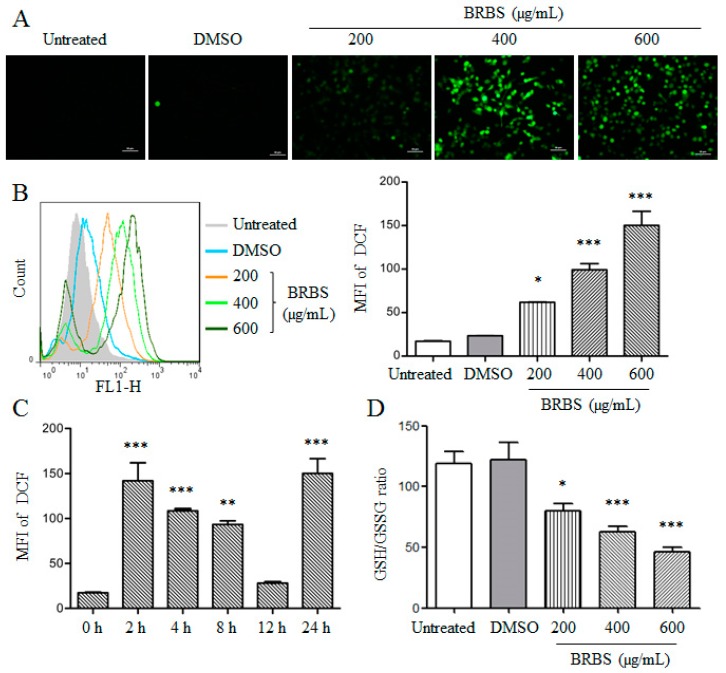
BRBS induces ROS generation in A549 cells. Cells were treated with different concentrations of BRBS for 24 h and stained with 2′,7′-Dichlorodihydrofluorescein diacetate (DCFH-DA). Samples were analyzed by inverted fluorescence microscopy (Scale bar = 50 µm) (**A**) and flow cytometry (**B**); (**C**) cells were treated with 600 µg/mL BRBS for 2, 4, 8, 12 and 24 h respectively, and stained with DCFH-DA. Samples were analyzed by flow cytometry; (**D**) cells were treated with BRBS for 24 h and the intracellular glutathione reduced/oxidized (GSH/GSSG) ratio was measured. * *p* < 0.05; ** *p* < 0.01; *** *p* < 0.001 compared to Untreated.

It has been reported that reactive oxygen species (ROS) are involved in mitochondria-dependent apoptosis due to mitochondria dysfunction [26,27]. After BRBS treatment for 24 h, A549 cells were stained with 2′,7′-Dichlorodihydrofluorescein diacetate (DCFH-DA) and observed by inverted fluorescence microscopy. The green fluorescence was enhanced by increasing of BRBS concentrations, as shown in Figure 5A. Furthermore, the ROS generation was detected by flow cytometry. We found that BRBS significantly increased intracellular ROS levels in a dose-dependent manner, as shown in Figure 5B. ROS production was also detected at indicated time points after 600 µg/mL BRBS treatment. The results showed that levels of ROS peaked at 2 h, decreased gradually from 4 h to 12 h and then increased at 24 h, as shown in Figure 5C. Glutathione (GSH) depletion is an early hallmark in ROS-mediated tumor cell apoptosis [28,29]. After BRBS treatment for 24 h, GSH/GSSG ratios in A549 cells were significantly decreased in a dose-dependent manner, as shown in Figure 5D. These results suggest that BRBS induced apoptosis by ROS induction and mitochondrial dysfunction in lung cancer cells.

### 2.5. BRBS Inhibits A549 Cell Migration

**Figure 6:**
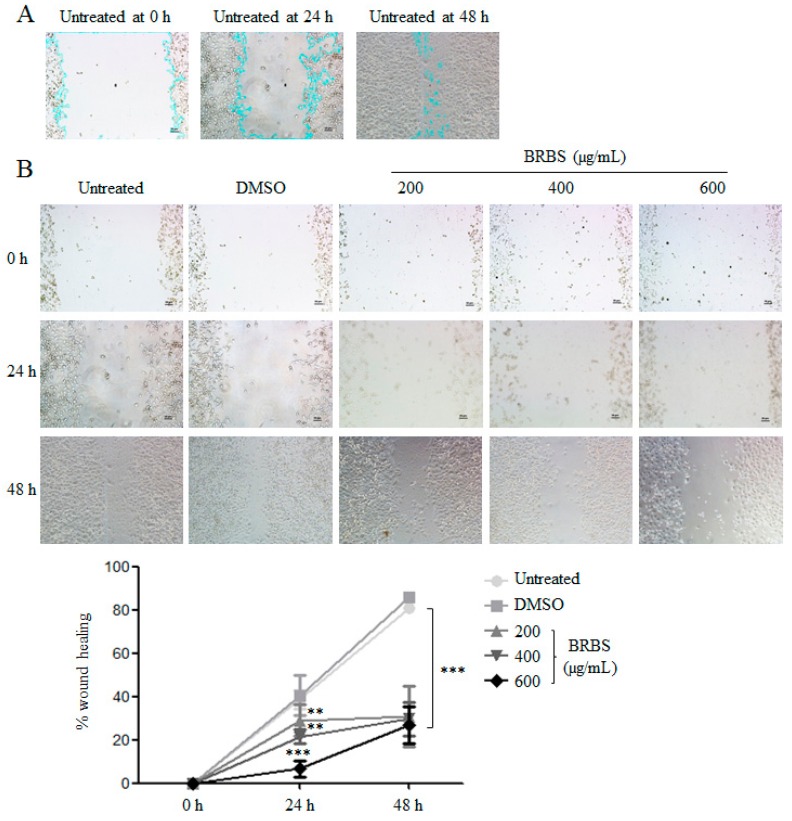
BRBS inhibits A549 cell migration (**A**) The gating strategy of scratch area by Image J. (**B**) After BRBS treatment for 24 and 48 h, A549 cell migration was observed by inverted microscope and analyzed by Image J. ** *p* < 0.01; *** *p* < 0.001 compared to Untreated.

The capacity for cancer cells to migrate is considered one of the critical processes in tumor metastasis. The effect of BRBS on A549 cell motility was evaluated by wound-healing assays. As shown in Figure 6, BRBS treatment significantly suppressed A549 cell migration in a dose-dependent manner, suggesting that BRBS might inhibit the invasion and metastasis of lung cancer.

## 3. Discussion

High dietary intake of *Brassica* vegetables is associated with a reduced risk of cancer and various compounds including flavonoids, lectin and 1-cyano-2,3-epithiopropane have been isolated from *Brassica* vegetables which can inhibit the growth of tumor cells through induction of apoptosis [30,31,32]. In the present study, we found that BRBS dose- and time- dependently suppressed the proliferation of A549 cells through induction of cell cycle arrest at G0/G1 phase and mitochondria-dependent apoptosis. A549 cell migration was also significantly inhibited by BRBS treatment.

ROS play an essential role in the regulation of cell cycle and apoptosis [33,34]. An active compound derived from *Brassica* vegetables, 3,3′-Diindolylmethane, induces cell cycle arrest and apoptosis in cancer cells through up-regulation of ROS production [35]. Similarly, we found that BRBS significantly increased intracellular ROS generation and decreased the intracellular GSH/GSSG ratio in A549 cells, which might mediate the induction of cell cycle arrest and apoptosis. ROS generation can activate c-Jun N-terminal kinase to promote and inhibit the expressions of pro-apoptotic and anti-apoptotic BCL-2 proteins respectively, which play a critical role in mitochondria-dependent apoptosis pathway through regulation of mitochondrial membrane integrity [34,36,37]. Upon BRBS treatment, the expressions of Bax and Bcl-2 in A549 cells are up-regulated and down-regulated respectively, which cause further reduction in MMP and the release of cytochrome c. Consequently, cytochrome c activates the processing of caspase-3 and PARP observed in this study, which finally induces the apoptosis in A549 cells.

In conclusion, BRBS inhibits the proliferation of A549 cells through induction of cell cycle arrest and mitochondria-dependent apoptosis. These results indicate that *B. rapa* L. might be considered as a functional vegetable with potential role for the treatment of lung cancer.

## 4. Materials and Methods

### 4.1. N-Butanol Subfraction of B. rapa L. (BRBS)

*B. rapa* L. was collected from Turpan in the Xinjiang Uygur Autonomous Region, China. Homogenate of fresh root of *B. rapa* L. was made and extracted twice using 10 volumes of water for 2 h at 60 °C. The water extraction was collected and centrifuged at 6000 rpm for 15 min. The pellet was collected and extracted with 10 volumes of 95% ethanol at 60 °C for three times according to the following order of extracting time: 3 h, 2 h and 1.5 h. The ethanol extraction was centrifuged at 6000 rpm for 15 min and the supernatant was collected and concentrated using a rotary vacuum evaporator at 45 °C. Then, the concentrated liquid was extracted with an equal volume of water saturated *n*-butanol. Finally, the *n*-butanol extraction was collected and dried at room temperature (RT). The dry *n*-butanol subfraction was dissolved in dimethyl sulfoxide (DMSO) (Sigma, St. Louis, MO, USA) at a concentration of 100 mg/mL and filtered with a 0.22 μm filter.

### 4.2. Cell Culture

The A549 cells were obtained from the Xinjiang Key Laboratory of Biological Resources and Genetic Engineering, Xinjiang University (Urumqi, Xinjiang, China) and maintained in GIBCO^®^1640 (Thermo Fisher Scientific, Waltham, MA, USA) supplemented with 10% fetal bovine serum (FBS) (MRC, Changzhou, China) and 1% Penicillin-Streptomycin (MRC) in a 5% CO_2_ atmosphere at 37 °C.

### 4.3. Cell Viability Assay

A549 cell (100 μL, 5 × 10^4^/mL) were seeded in a 96-well plate, cultured overnight and then treated with different concentrations (200, 400 and 600 μg/mL) of BRBS for 24, 48 and 72 h. Cisplatin (20 μg/mL) was used as a positive control. After centrifugation at 1200 rpm for 7 min, supernatant was discarded and 100 μL of MTT [3-(4,5-dimethyl-2-thiazolyl)-2,5-diphenyl-2-H-tetrazolium bromide] (Sigma) solution (0.5 mg/mL in PBS) was added to each well. After 4 h, 150 μL DMSO was added into each well to dissolve the formed formazan crystals. The optical density at 490 nm was measured by a 96-well microplate reader (Bio-Rad Laboratories, Hercules, CA, USA).

### 4.4. Observation of Cell Morphology

A549 cells were seeded in 96-well plates and incubated for 24 h at 37 °C. The cells were treated with different concentrations (200, 400 and 600 μg/mL) of BRBS for 24 h. After treatment, cell morphological changes were observed and photographed by inverted fluorescence microscopy (Nikon Eclipse Ti-E, Tokyo, Japan).

### 4.5. Determination of Intracellular Reactive Oxygen Species (ROS) and GSH/GSSG Ratio

A549 cells were treated with different concentrations (200, 400 and 600 μg/mL) of BRBS for 24 h or treated with 600 μg/mL of BRBS for 2, 4, 8, 12 and 24 h. After treatment, the level of intracellular ROS was measured by a ROS assay kit according to the manufacturer’s instructions (Beyotime, Shanghai, China). Briefly, cells were harvested and incubated with 2′,7′-dichlorofluorescein diacetate (DCFH-DA) for 20 min at RT. After washing with GIBCO^®^ 1640 3 times, the fluorescence intensity was determined by flow cytometry (BD FACSCalibur, San Jose, CA, USA) or inverted fluorescence microscopy.

After BRBS treatment for 24 h, GSH/GSSG ratios in A549 cells were spectrophotometrically measured using a GSH/GSSG ratio kit (Beyotime) according to manufacturer’s instructions.

### 4.6. Analysis of Apoptosis and Cell Cycle

A549 cells were treated with different concentrations (200, 400 and 600 μg/mL) of BRBS. After 24 h, cells were harvested, washed with phosphate-buffered saline (PBS) and stained with an Annexin V-FITC/propidium iodide (PI) Apoptosis Detection Kit (YEASEN, Shanghai, China) according to manufacturer’s instructions. For analysis of the cell cycle distribution, cells were harvested after BRBS treatment and fixed in cold 70% ethanol at 4 °C for 30 min. Then, cells were stained with PI for 30 min at 37 °C. All samples were analyzed by flow cytometry.

### 4.7. Detection of Ki-67

A549 cells were treated with different concentrations (200, 400 and 600 μg/mL) of BRBS. After 24 h, cells were harvested and washed with PBS. After washing, cells were fixed and permeabilized with Foxp3 Staining Buffer Set (eBioscience, San Diego, CA, USA) according to the manufacturer’s instructions. Intracellular staining was performed using FITC conjugated Ki-67 antibody (BD Biosciences, San Jose, CA, USA) for 15 min at RT. The samples were analyzed by flow cytometry.

### 4.8. Determination of Mitochondrial Membrane Potential

Mitochondrial membrane potential was determined by membrane-permeable JC-1 dye (Beyotime). Briefly, A549 cells were treated with BRBS, washed with PBS and stained with the JC-1 fluorescent probe according to manufacturer’s instruction for 20 min at RT. Samples were analyzed by flow cytometry and inverted fluorescence microscope.

### 4.9. Wound Healing Assay

A549 cells were seeded in 24-well plates at the concentration of 2 × 10^6^ cells per well and incubated at 37 °C overnight. Cell monolayers that converged almost 100% were wounded with a sterile 200 µL pipette tip. Detached cells were removed from the plates carefully with PBS and completed GIBCO^®^ 1640 was added. The cells were left either untreated or treated with different doses (200, 400 and 600 μg/mL) of BRBS. After 24 h, the medium was replaced with PBS and the scratched areas were photographed using inverted fluorescence microscopy. The scratch areas of samples were analyzed by Image J at the indicated time points. The percentage of wound healing was calculated by the equation: wound healing(%) = (1 − scratch area at indicated time point/scratch area at 0 h) × 100%.

### 4.10. Hoechst 33258 Staining

The morphological changes of A549 cell nuclei were analyzed by membrane-permeable DNA-binding dye Hoechst 33258. A549 cells were seeded into a 6-well plate at the concentration of 1 × 10^5^ cells/well and incubated overnight. The cells were treated with 400 and 600 μg/mL of BRBS for 24 h and fixed with 4% ice-cold paraformaldehyde at 4 °C for 10 min. After washing 3 times with PBS, cells were stained with Hoechst 33258 (Beyotime) at 4 °C for 10 min. Samples were observed by inverted fluorescence microscope.

### 4.11. Western Blot

After BRBS treatment, all adherent and floating cells were collected and whole cell lysates were prepared using RIPA Lysis Buffer (Beijing ComWin BiotechCo., Ltd., Beijing, China) on ice for 30 min. The protein concentration was determined using a bicinchoninic acid assay (BCA) Kit (Thermo Fisher Scientific) according to the manufacturer’s instructions. Each sample contained 35 µg protein was separated on 12% sodium dodecyl sulfate polyacrylamide gel electrophoresis (SDS-PAGE) and transferred to polyvinylidene difluoride (PVDF) membrane. After blocking with 5% skim milk, the membranes were incubated with respective primary antibodies (Cell Signaling Technology, Danvers, MA, USA) for 2 h at 37 °C. After washing with PBST solution (PBS with 0.05% Tween-20), the membranes were incubated with horse radish peroxidase (HRP)-conjugated secondary antibodies (Cell Signaling Technology) for 1 h at 37 °C. After washing 3 times with PBST, the target proteins were detected using an enhanced chemiluminescence (ECL) assay kit (Beyotime).

### 4.12. Statistical Analysis

All data were expressed as mean ± standard error of the mean (SEM). Statistical analysis was conducted using one-way analysis of variance (ANOVA). *p* < 0.05 was considered statistically significant.
